# Antiseptic Activity and Phenolic Constituents of the Aerial Parts of *Vitex negundo* var. *cannabifolia*

**DOI:** 10.3390/molecules15118469

**Published:** 2010-11-18

**Authors:** Tie-Jun Ling, Wei-Wei Ling, Yuan-Jun Chen, Xiao-Chun Wan, Tao Xia, Xian-Feng Du, Zheng-Zhu Zhang

**Affiliations:** Key Laboratory of Tea Biochemistry and Biotechnology of Ministry of Education and Ministry of Agriculture, Anhui Agricultural University, 130 West Changjiang Road, Hefei 230036, China; E-Mail: ling_tiejun@yahoo.com.cn (T.-J.L.)

**Keywords:** *Vitex negundo* var. *cannabifolia*, Verbenaceae, phenolics, antibacterial activities, spoilage microorganisms

## Abstract

Four phenolics, salviaplebeiaside (**1**), *γ*-tocopherol (**2**), chrysosplenol-D (**4**), and isovitexin (**5**), along with *α*-tocoquinone (**3**) and *β*-sitosterol (**6**) were isolated from the aerial parts of *Vitex negundo* var. *cannabifolia*. The isolation was performed using bio-assay tracking experiments. The structures of compounds **1**-**5** were established by spectroscopic means. The antibacterial activities of the compounds were assessed against *Escherichia coli*, *Bacillus subtilis*, *Micrococcus tetragenus* and *Pseudomonas fluorescens*. Chrysosplenol-D (**4**) exhibited activities against all the four spoilage microorganisms.

## 1. Introduction

*Vitex negundo* var. *cannabifolia* (Verbenaceae) is a shrub growing mainly in Yangzi River basin of China [1]. The plant was used as herbal medicine for the treatment of many diseases, such as colds, malaria, inflammation, sores and beriberi [2]. Some iridoids, lignans and other components in the species were reported in previous phytochemical studies [3,4,5]. The juice extracted from aerial parts of the plant has been used as a folk antiseptic in Anhui province of China for preventing meat from rotting. In this study, four phenolics (compounds **1**, **2**, **4** and **5**), along with two other compounds (**3** and **6**), were isolated and identified. Their antibacterial activities were examined and compound **4** was found to exhibit inhibitory activity against the four tested bacterial spp..

## 2. Results and Discussion

### 2.1. Antibacterial Activities of the Crude Extracts

The EtOH extract of the powdered dry aerial parts of *V. negundo* var. *cannabifolia* was successively fractionated with petroleum ether (PE), CHCl_3_ and *n*-BuOH. The *n*-BuOH fraction was further fractionated by Diaion HP-20 column chromatography (CC) using H_2_O, 50% EtOH and 95% EtOH in a sequential elution process to yield three fractions A-C. 

**Figure 1:**
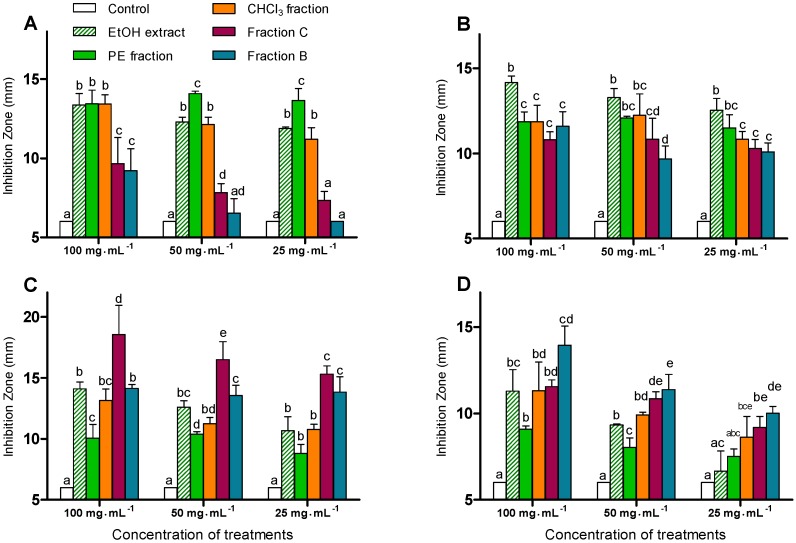
Antibacterial activities of the EtOH extract as well as PE, CHCl_3_, B and C fractions. Data are means ± SD, n = 3. Within each concentration treatment, columns containing the same letter are not significantly different according to Duncan’s new multiple range test at *P*<0.05. [(A) *E. coli*, (B) *B. subtilis*, (C) *M. tetragenus*, (D) *P. fluorescens*].

The antibacterial activities of the EtOH extract, as well as the PE, CHCl_3_, B, C fractions against *Escherichia coli*, *Bacillus subtilis*, *Micrococcus tetragenus*, and *Pseudomonas fluorescens* were evaluated by the hole plate diffusion method (see Experimental section 3.10 for more details) [6]. The EtOH extract and the four other fractions were individually dissolved and diluted with DMSO to obtain serial concentrations of 100, 50 and 25 mg·mL^-1^. The inhibition activities were evaluated by diameters of the inhibition zones (Figure 1). 

Figures 1A-D show the significant inhibitory activities of the crude extracts at all three tested concentrations. The activity against *E. coli* was enhanced as the polarity of the fractions decreased (Figure 1A), and the PE fraction showed significantly higher activity against *E. coli* at the concentrations of 50 and 25 mg·mL^-1^ versus the other fractions. However, there was no significant difference in the inhibition activity against *B. subtilis* among the four fractions (Figure 1B). The activity of fraction C against *M. tetragenus* was significantly higher than that of any other fraction (Figure 1C). Furthermore, at concentrations 50 and 25 mg·mL^-1^, both fractions C and B showed significantly higher activities compared to the PE and CHCl_3_ fractions. In Figure 1D, the inhibitory activities of the fractions against *P. fluorescens* were enhanced with the increase of the fractions’ polarity. Both fractions C and B displayed higher activities than the PE or CHCl_3_ fraction at all three concentrations. Overall fraction B showed a stronger inhibition against *P. fluorescens*. These results suggest that the bio-active components against *E. coli* were present in the lower-polarity (PE) fraction, while those against *M. tetragenus* and *P. fluorescens* were located in higher-polarity fractions (C and B).

### 2.2. Isolation and Identification of the Compounds

**Figure 2:**
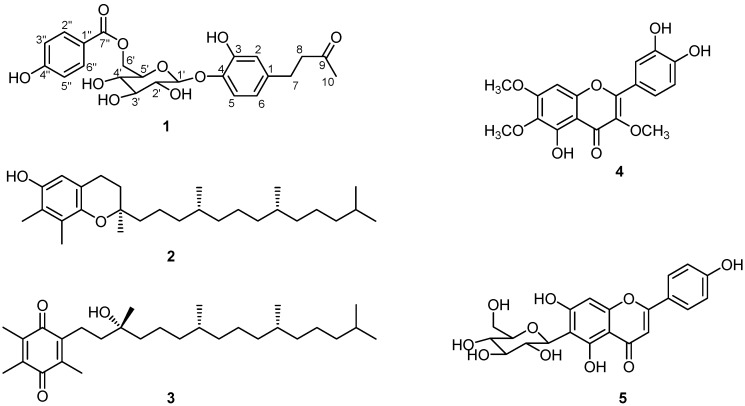
Structures of Compounds **1**-**5**.

The PE, B and C fractions were presumed to contain bio-active compounds from the results of the above assays, and were therefore separated by a combination of silica gel, ODS-A, and Sephadex LH-20 CC to yield compounds **1**-**6 **(Figure 2).

Compound **1** was obtained as a pale yellow gum. Its molecular formula was determined as C_23_H_26_O_10_ by the negative HRESI-TOF-MS signal at *m*/*z* 461.1462. The ^1^H-, ^13^C-NMR and HSQC spectra showed the signals of a carbonyl group (*δ*_C_ 211.13), a tertiary methyl group [*δ*_H_ 2.023 (3H, s), *δ*_C_ 30.00], a 1,2,4-trisubstituted benzene [*δ*_H_ 6.933 (1H, d, *J* = 8.4 Hz), *δ*_H_ 6.574 (1H, br s), and *δ*_H_ 6.284 (1H, dd, *J* = 8.4, 1.6 Hz)], and a *β*-D-glucopyranosyl moiety [*δ*_H_ 4.625 (1H, d, *J* = 5.2 Hz), *δ*_C_ 104.30, 77.59, 75.83, 74.88, 72.07, 64.83]. The high field region of the ^1^H-NMR spectrum gave a 4H singlet (*δ*_H_ 2.617), which was correlated with two carbon signals at *δ*_C_ 45.81 and 30.20 in the HSQC spectrum. The above information was characteristic of a phenylbutanone glucoside, containing a glucopyranosyl moiety, and an aromatic ring (A-ring) with a butanone substituent [7]. The 4H singlet (C**H_2_**-7 and C**H_2_**-8) mentioned above was believed to be the results of the magnetic equivalence of those protons. The ^1^H- and ^13^C-NMR spectra of **1** were very similar to those of vitexfolin C, which hads a *p*-hydroxybenzoyl group (B-ring) at its 2′ position, and by careful comparison of the spectral data of **1** with those of vitexfolin C [7], the presence of a *p*-hydroxybenzoyl group [*δ*_H_ 7.753 (2H, d, *J* = 8.8 Hz), *δ*_H_ 6.648 (2H, d, *J* = 8.8 Hz), *δ*_C_ 168.14 (C-7″), 165.63 (C-4″), 133.03 (C-2″, 6″), 120.95 (C-1″), 116.82 (C-3″, 5″)] in **1** could indeed be readily deduced. The key differences between the NMR spectra of **1** and those of vitexfolin C were the chemical shifts of the ^1^H- and ^13^C-NMR signals of the 2′, 5′ and 6′ positions. In the NMR spectra of **1**, the chemical shifts of H-2′, C-2′ and C-5′ were upshifted to *δ*_H_ 3.446, *δ*_C_ 74.88 and *δ*_C_ 75.83 ppm, respectively, while those of H-5′, H-6′a, H-6′b and C-6′ were downshifted to *δ*_H_ 3.641, *δ*_H_ 4.575, *δ*_H_ 4.275, and *δ*_H_ 64.83 ppm, respectively, suggesting that the *β*-D-glucopyranosyl moiety in **1** was 1′,6′-disubstituted instead of 1′,2′-disubstituted as in vitexfolin C. By analysis on the reported phenylbutanone glucosides in *Vitex* spp., **1** was assumed to be a 1′-(A-ring),6′-(B-ring)-disubstituted glucopyranose [7]. The hypothesis was confirmed by analysis of the HMBC spectrum, which showed cross peaks between H-6′ (*δ*_H_ 4.575 and *δ*_H_ 4.275) and C-7″ (*δ*_C_ 168.14), as well as between H-1′ (*δ*_H_ 4.625) and C-4 (*δ*_C_ 144.99). On the basis of this evidence, **1** was identified as 4-{4-*O*-[6-(4-hydroxybenzoyl)-*O*-*β*-D-glucopyranosyl]-3-hydroxyphenyl}-2-butanone. The structure was previously reported as salviaplebeiaside from *Salvia plebeian* [8]. 

By comparison their spectroscopic data (see the Experimental section) with those reported in the references, compounds **2**-**5** were identified as *γ*-tocopherol, *α*-tocoquinone, chrysosplenol-D and isovitexin, respectively [9,10,11,12]. Compound **6** were identified as *β*-sitosterol by comparing its Rf value with that of an authentic sample in a TLC experiment.

### 2.3. Antibacterial Activities of Compounds 1-5

The antibacterial activities of **1**-**5** against *E. coli*, *B. subtilis*, *M. tetragenus*, and *P. fluorescens* were also evaluated by the hole plate diffusion method. Compound **4** exhibited weak activities against *E. coli*, *B. subtilis* and *M. tetragenus*, with minimal inhibitory concentration (MIC) values of 500, 500 and 250 μg·mL^-1^, respectively. At the same concentrations, ampicillin sodium displayed much stronger inhibition against the corresponding microorganisms. Nevertheless, the inhibitory activity of **4** against *P. fluorescens* was comparable with that of ampicillin sodium (MIC, 500 μg·mL^-1^, see Table 2).

## 3. Experimental

### 3.1. General

IR spectra were obtained on a Nicolet 8700 FT-IR spectrophotometer (Thermo, USA). The ^1^H-NMR (400 MHz), ^13^C-NMR (100 MHz) and 2D-NMR spectra such as HSQC and HMBC were recorded on a Bruker Avance-400 (Bruker, Switzerland). Chemical shifts are expressed in ppm (*δ*). The ESI-MS data were obtained with a Finnigan LTQ LC/MS system (Thermo, USA) by direct injection. The HRESI-TOF-MS was recorded on an Agilent 6210 instrument (Agilent Technologies, Inc., USA) by direct injection. Silica gel 60 (200-300 mesh, Qingdao Marine Chemical Co. Ltd., Qingdao, PR China) and YMC GEL ODS-A (50 μm, YMC Co. Ltd., Japan) were used for CC. MeOH was used as the eluant in Sephadex LH-20 CC. TLC was performed using precoated silica gel plates (GF254, Liangchen Chemical Co. Ltd., Huoshan, Anhui province, PR China), or precoated RP-18 silica gel plates (F254, Merck, Germany), with detection by spraying with 10% H_2_SO_4_/ethanol reagent followed by heating.

### 3.2. Plant Material

Aerial parts of *V.* *negundo* var. *cannabifolia* were collected in the rural area of Xuan-Cheng, Anhui Province, China, in summer of 2008, and identified by Prof. Sheng-Ni Tian, Anhui Agricultural University, and Prof. Yue-Hong Yan, Hunan University of Science and Technology. A voucher specimen was deposited at the Laboratory of Botany, School of Life Sciences, Anhui Agricultural University.

### 3.3. Extraction

Dry stems and leaves of *V.* *negundo* var. *cannabifolia* (3.0 kg) were ground to a fine powder, which were extracted with 95% EtOH three times (24, 48 and 48 h with 10 L each time) by percolation at room temperature. The EtOH percolate was concentrated *in vacuo* to a syrup (EtOH extract, 420 g). This syrup was suspended in H_2_O and the aqueous suspension (1000 mL) was successively extracted with PE (500 mL, 3 times), CHCl_3_ (500 mL, 3 times), and *n*-BuOH (500 mL, 3 times) at room temperature. The PE and the CHCl_3_ extracts, on concentration, yielded a dark green syrup (PE fraction, 60 g) and a brown syrup (CHCl_3_ fraction, 40 g) respectively. The *n*-BuOH extraction, upon concentration under reduced pressure, afforded 90 g of a brown syrup. This syrup was further fractionated by Diaion HP-20 CC using H_2_O, 50% EtOH and 95% EtOH in a sequential elution process, yielding three fractions A-C.

### 3.4. Isolation of the Compounds

The PE fraction was subjected to CC on silica gel, eluted with PE-EtOAc mixtures of increasing polarity (from 15:1 to 8:1), to give three fractions (PE1-PE3). Fraction PE1 was further subjected sequentially to silica gel CC using 99:1 PE-EtOAc as eluant, and to ODS-A CC using methanol as eluant, to afford **2** (13 mg), **3** (21 mg) and **6** (60 mg). Fraction B (53 g) was subjected sequentially to silica gel CC eluted with CH_2_Cl_2_-CH_3_OH mixtures of increasing polarities (from 10:1 to 5:1), to ODS-A CC using CH_3_OH-H_2_O (2:3) as eluant, and to Sephadex LH-20 CC to afford **1** (27 mg) and **5** (10 mg). Fraction C (4 g) was subjected to silica gel CC eluted with CH_2_Cl_2_-MeOH (10:1) and repeated Sephadex LH-20 CC to yield **4** (24 mg).

### 3.5. Salviaplebeiaside *(**1**)*

Pale yellow gum. IR (KBr) *ν*_max_ (cm^–1^): 3,420, 2,923, 1,702, 1,609, 1,594, 1,511, 1,448, 1,281, 1,167, 1,073, 882, 852, 800, 772, 729 and 698. HRESI-TOF-MS: *m/z* 461.1462 [M – H]^–^ (calcd. for C_23_H_25_O_10_^–^, 461.1453) in negative mode.^ 1^H- and ^13^C-NMR data, see Table 1.

**Table 1:** ^1^H- and ^13^C-NMR data of **1** (in methanol-*d*_4_).

Position	*δ*_H_ (*J* in Hz)	*δ*_C_		Position	*δ*_H_ (*J* in Hz)	*δ*_C_
1	─	138.13		3′	3.446 *^b^*	77.59
2	6.574 br s	117.13		4′	3.357 m	72.07
3	─	148.38		5′	3.641 m	75.83
4	─	144.99		6′a	4.575 br d (11.6)	64.83
5	6.933 d (8.4)	118.89		6′b	4.275 dd (11.6, 7.6)	
6	6.284 dd (8.4, 1.6)	120.34		1″	─	120.95
7	2.617 br s *^a^*	30.20		2″	7.753 d (8.8)	133.03
8	2.617 br s *^a^*	45.81		3″	6.648 d (8.8)	116.82
9	─	211.13		4″	─	165.63
10	2.023 s	30.00		5″	6.648 d (8.8)	116.82
1′	4.625 d (5.2)	104.30		6″	7.753 d (8.8)	133.03
2′	3.446 *^b^*	74.88		7″	─	168.14

*^a, b^* signals were overlapped.

### 3.6. γ-Tocopherol *(**2**)*

Yellow oil; ESI-MS *m/z* 417 [M + H]^+^, 415 [M – H]^–^; ^1^H-NMR (CDCl_3_) *δ*: 6.372 (1H, s, H-5), 2.669 (2H, m, H-4), 2.136, 2.113 (each 3H, s, H-7a, 8a), 1.243 (3H, s, H-2a), 0.872-0.846 (12H, m, H-4′a, 8′a, 12′a, 13′); ^13^C-NMR (CDCl_3_) *δ*: 146.3 (C-6), 145.9 (C-8a), 125.9 (C-8), 121.7 (C-7), 118.4 (C-4a), 112.3 (C-5), 75.6 (C-2), 40.2 (C-1'), 39.5 (C-11'), 37.5 (C-3', 5', 7', 9'), 32.9 (C-4', 8'), 31.6 (C-3), 28.0 (C-12'), 24.9 (C-10'), 24.5 (C-6'), 24.2 (C-2a), 22.8 and 22.7 (C-12'a and C-13'), 22.4 (C-4), 21.1 (C-2'), 19.8 (C-4'a, 8'a), 11.9 (C-7a, 8a).

### 3.7. α-Tocoquinone *(**3**)*

Yellow oil; ESI-MS *m/z* 447 [M + H]^+^, 445 [M – H]^–^; ^1^H-NMR (CDCl_3_) *δ*: 2.539 (2H, m, H-1'), 2.032 (3H, s, H-3a), 2.003 (6H, s, H-2a, 5a), 1.228 (3H, s, H-3'a), 0.867-0.830 (12H, m, H-4′a, 8′a, 12′a, 13′); ^13^C-NMR (CDCl_3_) *δ*: 187.8 (C-4), 187.3 (C-1), 144.5 (C-6), 140.6 (C-5), 140.5 (C-3), 140.3 (C-2), 72.8 (C-3'), 42.4 (C-4'), 40.3 (C-2'), 39.4 (C-14'), 37.7 (C-6'), 37.5 (C-10'), 37.4 (C-12'), 32.7 (C-7', 11'), 29.7 (C-8'), 27.9 (C-15'), 26.7 (Me-3'), 24.9 (C-13'), 24.6 (C-9'), 22.8 (Me-15'), 22.7 (Me-15'), 21.5 (C-1'), 21.4 (C-5'), 19.8 (Me-7'，11'), 12.5 (Me-2), 12.4 (Me-2), 11.9 (Me-5).

### 3.8. Chrysosplenol-D *(**4**)*

Yellow amorphous powder; ESI-MS *m/z* 361 [M + H]^+^, 383 [M + Na]^+^, 359 [M – H]^–^; ^1^H-NMR (DMSO-*d*_6_) *δ*: 7.56 (1H, d, *J* = 2.1 Hz, H-2'), 7.45 (1H, dd, *J* = 8.4, 2.1 Hz, H-6'), 6.91 (1H, d, *J* = 8.4 Hz, H-5'), 6.80 (1H, s, H-8), 3.93 (3H, s, 7-OCH_3_), 3.81 (3H, s, 3-OCH_3_), 3.76 (3H, s, 6-OCH_3_); ^13^C-NMR (DMSO-*d*_6_) *δ*: 178.1 (C-4), 158.5 (C-7), 156.0 (C-9), 151.7 (C-5), 151.5 (C-2), 149.3 (C-4'), 145.4 (C-3'), 137.6 (C-3), 131.5 (C-6), 120.6 (C-1'), 120.3 (C-6'), 115.7 (C-2'), 115.3 (C-5'), 105.5 (C-10), 91.2 (C-8), 60.0, 59.6.3 (6, 3-OCH_3_), 56.4 (7-OCH_3_).

### 3.9. Isovitexin *(**5**)*

Yellow amorphous powder; ESI-MS *m/z* 455 [M + Na]^+^, 431 [M – H]^–^;^ 1^H-NMR (CD_3_OD) *δ*: 7.71 (2H, d, *J* = 8.8 Hz, H-2', 6'), 6.81 (2H, d, *J* = 8.8 Hz, H-3', 5'), 6.47 (1H, s, H-8), 6.38 (1H, s, H-3), 4.81 (1H, d, *J* = 10 Hz, H-1"), 4.07 (1H, dd, *J* = 9.6, 9.2 Hz, H-2"), 3.78 (1H, dd, *J* = 12.0, 2.0 Hz, H-6"a), 3.65 (1H, dd, *J* = 12.0, 5.2 Hz, H-6"b), 3.40-3.29 (3H, m, H-3", 4", 5"); ^13^C-NMR (CD_3_OD) *δ*: 184.0 (C-4), 166.2 (C-2), 165.0 (C-7), 162.8 (C-4'), 162.0 (C-5), 158.7 (C-9), 129.4 (C-2', 6'), 123.1 (C-1'), 117.0 (C-3', 5'), 109.2 (C-6), 105.2 (C-10), 103.9 (C-3), 95.3 (C-8), 82.6 (C-5"), 80.2 (C-3"), 75.3 (C-1"), 72.6 (C-2"), 71.8 (C-4"), 62.9 (C-6").

### 3.10. Anti-bacterial Assays of the Crude Extracts

Anti-bacterial activities were evaluated by the hole plate diffusion method [6]. The test microorganisms were *E. coli*, *B. subtilis*, *M. tetragenus*, and *P. fluorescens*, which were obtained from the School of Basic Medical Sciences, Anhui Medical University, Hefei, P.R. China. The EtOH extract, PE and CHCl_3_ fractions, as well as fractions B and C were individually dissolved and diluted with DMSO to obtain serial concentrations of 100, 50 and 25 mg·mL^-1^. Six mm wide holes were bored with a sterilized steel borer into the Nutrient Agar Media (beef extract 3 g, peptone 10 g, agar 17 g, NaCl 5 g, H_2_O 1,000 mL, pH 7.2) in the Petri dish inoculated with the test microorganism. The solution of the compound (60 μL) at a specific concentration was added into each of the holes. DMSO was used as the negative control. The plates were then incubated at 37 °C for 24 hours. The diameters of the inhibition zones were measured and recorded. The assays were performed three times in order to guarantee reproducibility of results (see Figure 1).

### 3.11. Anti-bacterial Assays of the Compounds

Compounds **1-5 **and ampicillin sodium (positive control) were individually dissolved and diluted with DMSO to obtain serial concentrations of 1000, 500, 250, 125 and 62.5 μg·mL^-1^ (for ampicillin sodium, the solutions were serially diluted from 1000 to 0.03 μg·mL^-1^). The anti-bacterial assays were also performed by the hole plate diffusion method as described above. The inhibition zones around the holes were measured and the MIC, which was defined as the lowest concentration being able to inhibit any visible bacterial growth, was recorded. The assays were performed three times for statistical analysis (see Table 2).

### 3.12. Statistical Analysis

The data in Figure 1 are presented as means ± SD. The values were evaluated by one-way analysis of variance (ANOVA), followed by Duncan’s multiple range tests using GraphPad Prism 5.0 software (GraphPad Software Inc., San Diego, CA, USA). Differences were considered significant at *P* < 0.05.

**Table 2:** Antibacterial Activities of Compounds **1**-**5**.

Compd.		MIC (μg·mL^-1^) *^a^*			
		*E. coli*	*B. subtilis*	*M. tetragenus*	*P. fluorescens*
**1**		>1000	>1000	>1000	>1000
**2**		>1000	>1000	>1000	>1000
**3**		>1000	>1000	>1000	>1000
**4**		500	500	250	500
**5**		>1000	>1000	>1000	>1000
AMP *^b^*		0.122	0.061	0.244	250

*^a^*: The results were the average of three readings; *^b^*: ampicillin sodium.

## 4. Conclusions

This is the first report on the isolation of salviaplebeiaside, *γ*-tocopherol and *α*-tocoquinone from the genus *Vitex*. Compounds **1**-**5** were reported from investigated species for the first time. The apoptosis-inducing and antimalarial activities, as well as vascular relaxation effects of **4** had been reported [13,14,15,16]. With respect to antibacterial activities, the inhibition effects of **4** on *Staphylococcus aureus*, *Cladosporium cucumerinum* and *Bacillus cereus* were documented [11,17]. Our study indicated the inhibition activities of **4** on four spoilage microorganisms for the first time. It is known that *P. fluorescens* plays an important role in rotting of meat. The results of the present assays suggest chrysosplenol-D could be used as a potential antiseptic food additive. Furthermore, this compound might also be one of the key components to account for the medicinal usage of the plant. 

In our previous work, the compositions of the essential oil from the aerial parts of *V.negundo* var. *cannabifolia* and their antiseptic activities were analyzed [18]. However, neither the essential oil, nor the compounds isolated from the PE fraction displayed any significant inhibitory activity against *P. fluorescens*. 

A characteristic of phenolic-rich high-polar fraction in the fruits of the investigated species was revealed by previous phytochemical studies [5]. Our work also revealed that phenolics were the predominant components in the high-polar fraction of the aerial parts of the plant. As the high-polar fraction was confirmed as the significant antiseptic fraction by our bio-assays, the phenolics could be the key antiseptic constituents of *V.negundo* var. *cannabifolia*, even though more chemical examinations have to be done for this plant.
